# Clinical study of children with Takayasu arteritis: a retrospective study from a single center in China

**DOI:** 10.1186/s12969-017-0164-2

**Published:** 2017-04-17

**Authors:** Ye Feng, Xuemei Tang, Mingyue Liu, Juan Zhou, Xiaodong Zhao, Qiu Li

**Affiliations:** 1 Department of Rheumatology and Immunology, Children’s Hospital of Chongqing Medical University, Ministry of Education Key Laboratory of Child Development and Disorders, No. 136, Zhongshan 2nd Road, Yuzhong District, Chongqing, 400014 People’s Republic of China; 2 China International Science and Technology Cooperation base of Child development and Critical Disorders, No. 136, Zhongshan 2nd Road, Yuzhong District, Chongqing, 400014 People’s Republic of China; 3 Chongqing Key Laboratory of Pediatrics, No. 136, Zhongshan 2nd Road, Yuzhong District, Chongqing, 400014 People’s Republic of China

**Keywords:** Pediatric rheumatology, Takayasu’s arteritis, Diagnostic framework, Hypertension, Ultrasonography, Disease activity score

## Abstract

**Background:**

Delayed diagnosis of childhood Takayasu arteritis (TA) is common due to its atypical symptoms. The objective of the present study was to summarize the clinical features of childhood TA to raise awareness and improve management.

**Methods:**

Eleven children diagnosed with TA at our hospital were enrolled. Clinical information, diagnosis, treatment, and outcome were then examined retrospectively. The Pediatric Vasculitis Activity Score (PVAS) and the Indian Takayasu Clinical Activity Score (ITAS2010) were used to assess disease activity.

**Results:**

Male-to-female ratio was 4:7. The mean age was 9.4 (1.4–14) years and the average time to diagnosis was 40.6 days (12–90 days). All patients suffered from hypertension and few had immunologic abnormalities. Two patients had low levels of autoantibodies and one had elevated immunoglobulin E levels. Aberrant (elevated) laboratory parameters included erythrocyte sedimentation rate (ESR) (9/10 patients, 90.0%), protein excretion (8/9 patients, 88.9%), renin-angiotensin-aldosterone system (RAAS) activity (5/5 patients, 100.0%), and serum lipid levels (3/5 patients, 60%). The common onset patterns were headache with convulsions (27.2%) and kidney damage (27.2%). The abdominal aorta (81.8%) and renal artery (72.7%) were the most commonly involved vessels. At presentation, the mean PVAS and ITAS2010 scores were 12.1 (6–26)/63 and 9.7 (5–14)/57, respectively. All patients were treated with glucocorticoids and antihypertensive agents; two underwent renal artery stent placement.

**Conclusion:**

The diagnosis of TA should be considered in patients with pediatric hypertension and high expression of inflammatory markers or abnormal urine results. Doppler ultrasonography of major vessels may be helpful. PVAS and ITAS2010 both help to evaluate disease activity, and the PVAS is recommended for patients with kidney damage. Glucocorticoid and antihypertensive agents are effective. Interventional therapy can be an option for patients with persistent hypertension.

**Electronic supplementary material:**

The online version of this article (doi:10.1186/s12969-017-0164-2) contains supplementary material, which is available to authorized users.

## Background

Takayasu arteritis (TA) is a chronic type of systemic large vessel vasculitis, mainly involving the aorta and its main branches. Early symptoms include systemic inflammation and ischemia of involved organs [1].

Unfortunately, diagnosis of childhood TA is often delayed, particularly in children under 10 years old, a factor that contributes to cardiovascular damage and mortality [2, 3]. Therefore, many studies have attempted to identify new technologies that are both reliable and sensitive, for example, ^18^F-fluorodeoxyglucose (^18^F-FDG) positron emission tomography-computed tomography (PET-CT) [4]. Although such technologies have made diagnosis more timely and accurate, the most important factor that will yield an early diagnosis is an improvement in doctors’ clinical thinking and selection of the relevant tests/examinations.

Here, we performed a retrospective analysis of children diagnosed with TA at a single Chinese center and summarize the clinical features and follow-up data. The aim is to help clinicians reach an early diagnosis and to improve the management of chronic vasculitis.

## Methods

The study enrolled 11 children diagnosed with TA at our hospital from 2000 to 2015. Demographic data, clinical manifestations, laboratory and imaging results, diagnostic and therapeutic processes, and outcomes were then analyzed retrospectively. All patients were followed up via regular clinic visits or by telephone interview.

### Diagnostic criteria

Patients were assessed according to the 2008 EULAR/PRINTO/PRES criteria [5], which include angiographic abnormality (conventional, CT, or MRI) of the aorta or its main branches and pulmonary arteries (mandatory criterion) plus at least one of the following: (1) absence of the peripheral artery pulse or claudication induced by physical activity; (2) a >10 mm Hg difference in systolic BP in all four limbs; (3) Bruits over large arteries; (4) hypertension (when compared with age-matched healthy children); and (5) increased levels of acute phase reactants (erythrocyte sedimentation rate(ESR) and/or C reactive protein(CRP). Fibromuscular dysplasia or similar causes were excluded.

### Evaluation of disease activity

Two tools were used for retrospective evaluation: the Pediatric Vasculitis Activity Score (PVAS) and the Indian Takayasu Clinical Activity Score (ITAS-2010) [6, 7]. The PVAS is derived from the Birmingham Vasculitis Activity Score (BVAS) and is specific for pediatric vasculitis. It assesses new or worsening features occurring during the last 4 weeks or symptoms that have persisted for 3 months. It is comprised of nine sections with a total score of 63. The ITAS-2010 is a tool specific for Takayasu arteritis and comprised of six organ-based systems. It evaluates new symptoms or symptoms that have worsened during the last 3 months, the maximum score is 51. The ITAS-A score is equivalent to the ITAS-2010 score plus the acute phase reactant (ESR and CRP) scores, which increases the maximum score to 57 [6, 7]. The brief comparison between PVAS and ITAS-2010 showed in Additional file 1 (not in the text).

### Evaluation of treatment effects

A treatment was deemed effective if it resulted in improved clinical symptoms, a reduction in blood pressure, no radiographic progression, or a reduction in disease activity scores.

### Data management

Statistical analysis was performed using SPSS version 19.0. Data were expressed as the median or mean, and the significance of differences was evaluated using Student’s *t*-test or the Mann-Whitney *U*-test as appropriate. Spearman’s rank correlation analysis was used to examine associations between the PVAS and ITAS.

## Results

### General data

The TA group was comprised of four boys (36.4%) and seven girls (63.6%). The mean age of disease onset was 9.4 ± 3.9 years (range, 1.4–14 years). One of the eleven was 1.4 and younger than 5 years of age, four were aged 5–10 years, and six were aged over 10 years. Girls showed a later age of onset (11.3 ± 1.7 years) than boys (6.1 ± 4.1 years) (*p* < 0.05).

The mean time to diagnosis was 40.6 ± 21.6 days (range, 12–90 days), with a mean of 59.6 days for boys and 29.6 days for girls (*p* < 0.05). None of the patients had a family history of vasculitis.

### Clinical features

All 11 children had hypertension (nine at the first clinic visit and two at follow-up). The common manifestations are listed in Table 1.

**Table 1:** Clinical manifestations

Symptom	Number (%)	
Hypertension	11	(100%)
Fever	5	(45.4%)
BP discrepancy	5	(45.4%)
Vomiting	5	(45.4%)
Abdominal pain	4	(36.4%)
Headache	3	(27.2%)
Convulsion	3	(27.2%)
Bruits	3	(27.2%)
Weak pulse	3	(27.2%)
Abnormal urine output	3	(27.2%)

According to the above data, six onset patterns can be summarized. The most common patterns were headache with convulsions and kidney damage, with three patients showing each of these patterns. Table 2 shows the onset pattern, initial presentation, and PVAS/ITAS-2010/ITAS-A scores at the first clinic visit.

**Table 2:** Onset pattern, initial presentation, and disease activity scores

Serial number	Age of initial presentation	Gender	Onset pattern	Initial presentation	PVAS/ITAS/ITAS-A score at first clinic
1	12.8y	M	Cardiac failure	Shortness of breath and chest tightness with pink frothy sputum	10/5/6
2	5.3y	M	Headache with convulsions	Headache, convulsions, and intermittently febrile	11/7/8
3	5.0y	M	Headache with convulsions	Headache, convulsions, and fever	17/14/15
4	10.8y	F	Kidney damage	Dizziness, chest tightness, and palpitations	20/13/18
5	1.4y	M	Kidney damage	Macro-albuminuria	10/6/6
6	12.4y	F	Kidney damage	Precordial distress, vomiting, and oliguria	18/7/-
7	9.4y	F	Fever	Prolonged fever	6/5/8
8	9.2y	F	Hypertension	Hypertensive at the preoperative physical examination; Symptoms of Henoch-Schönlein Purpura	6/7/7
9	12.7y	F	Numbness of limbs	Numbness of the right limbs	18/11/17
10	10.7y	F	Hypertension	Hypertensive on physical examination	14/10/12
11	14.0y	F	Headache with convulsions	Cough, headache, convulsions, and fever	11/8/12

In addition to TA, patient no. 1 had multiple lung cysts, patient no. 8 had Henoch-Schönlein Purpura, and patient no. 7 had autoimmune hemolytic anemia at the same time.

### Laboratory and imaging results

Immune screening was almost normal, with only two patients (22.2%) showing low levels of autoantibodies (nine patients were tested). One patient had high serum IgE levels (10%; 10 patients tested), and seven tested patients were negative for anti-neutrophil cytoplasmic antibodies (ANCA). Inflammatory markers and other laboratory results are shown in Table 3.

**Table 3:** Laboratory test results for TA children

Test	Number (%)	
Elevated ESR	9/10	(90%)
Elevated CRP	3/10	(30%)
Elevated renin	5/5	(100%)
Elevated serum lipids	3/5	(60%)
Increased neutrophil percentage	5/11	(45.5%)
Low hemoglobin level	4/11	(36.4%)
Hypokalemia	4/11	(36.4%)
24H protein excretion	8/9	(88.8%)
Urine routine	6/11	(54.5%)
Autoantibodies	2/9	(22.2%)

All five tested patients showed increased renin levels, and four of nine patients tested showed evidence of prior tuberculosis infection: two were positive on sputum testing and imaging, whereas two were suspected cases (only the interferon-gamma release test was positive).

Computed tomographic angiography (CTA) revealed thickening of the arterial wall, along with lumen narrowing and vessel dilatation. Patient no. 1 showed radiographic evidence of aortic dissection. Ultrasonography revealed a rough inner wall or increased blood flow.

The abdominal aorta (nine patients, 81.8%) and renal artery (eight patients, 72.7%) were most frequently affected; The details are presented in Table 4.

**Table 4:** Involved vessels

Involved vessel	Number of patients (%)		Involved vessel	Number of patients (%)	
Ascending aorta	1	(9.1%)	Abdominal aorta and its branches:	11	(100%)
Aortic arch and its branches:	5	(45.4%)	Abdominal aorta	9	(81.8%)
Aortic arch	3	(27.2%)	Celiac trunk	5	(45.4%)
Brachiocephalic trunk	2	(18.2%)	Superior mesenteric artery	4	(36.4%)
Common carotid artery	3	(27.2%)	Inferior mesenteric artery	1	(9.1%)
Subclavian artery	2	(18.2%)	Renal artery	8	(72.7%)
Radial artery	1	(9.1%)	Iliac artery	1	(9.1%)
Intracranial artery	2	(18.2%)	Femoral artery	1	(9.1%)
Thoracic aorta	3	(27.2%)	Coronary artery	1	(9.1%)
			Pulmonary artery	2	(18.2%)

Four of the eight patients with an involved renal artery also showed unilateral renal atrophy. The four patients with superior mesenteric artery involvement suffered no ischemic abdominal pain. Among the two patients showing intracranial artery involvement, patient no. 4 (anterior cerebral artery) felt dizzy and patient no. 9 (middle and posterior cerebral artery) suffered transient amaurosis. Two patients had pulmonary artery involvement, although none had pulmonary hypertension or pulmonary dysfunction. We identified two patients (of three patients examined) with funduscopic abnormalities (no. 4 and no. 8), although both had normal visual acuity.

According to the new angiographic classification of TA (International Conference of Takayasu’s arteritis in Tokyo, 1994) [8], the most common type of vessel involvement was type V (six patients; 45.5%), followed by type IV (four patients; 36.45%). Two patients showed type IIa and type III involvement, and none showed type I or type IIb involvement (Additional file 2).

### Onset patterns and diagnostic process

**Fig. 1:**
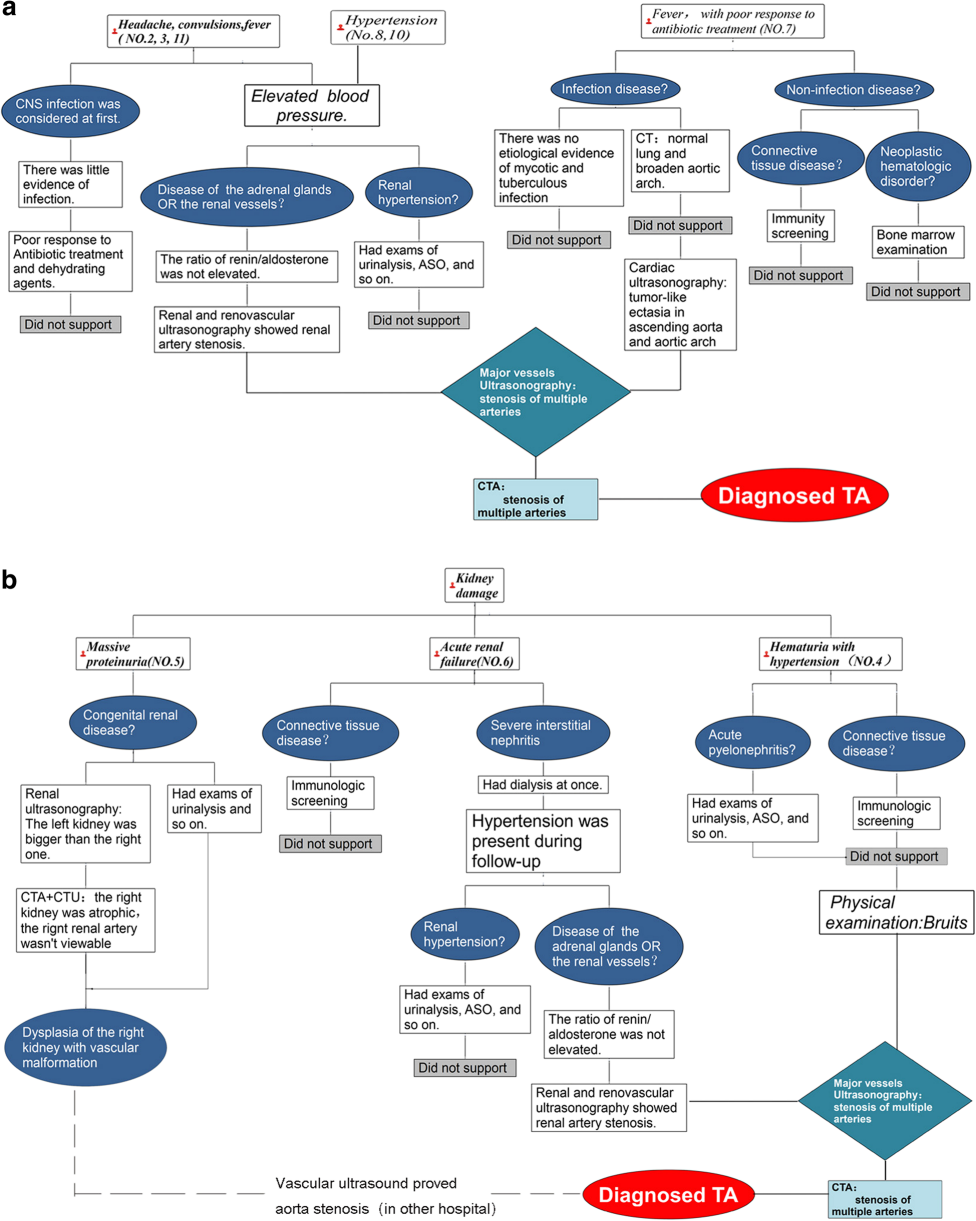
Diagnostic procedure and clinical framework of TA in cohort. Legend: **a** The onset patterns of headache with convulsions, Hypertension as well as fever. **b** The onset patterns of kidney damage

The diagnostic and analysis framework for each onset pattern is shown in Fig. 1a and b.

The example in Fig. 1a shows that the doctor firstly considered an intracranial infection in a child presenting with headache, convulsions, and fever. However, there was little evidence to support this diagnosis. Then doctors recognized that hypertension was a prominent symptom and carried relevant examinations. Further findings upon vessel ultrasonography and CTA enabled a final diagnosis.

### Disease activity scores

**Fig. 2:**
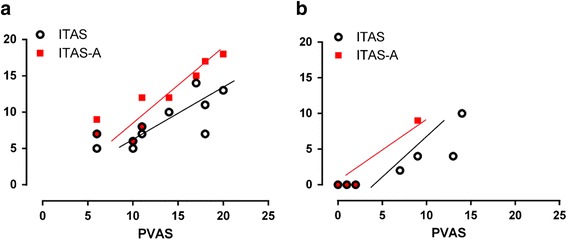
Correlation between Takayasu arteritis disease activity measures. Legend: **a** Correlation at time of diagnosis: ITAS-2010, r1 = 0.77, *P* = 0.002; ITAS-A, r2 = 0.84, *P* = 0.002. **b** Correlation at time of latest follow-up: ITAS-2010, r1 = 0.92, *P* < 0.001; ITAS-A, r2 = 0.72, *P* > 0.05. PVAS, Pediatric Vasculitis Activity Score; ITAS-2010, Indian Takayasu Arteritis Activity Score; ITAS-A, Indian Takayasu Arteritis Activity Score (containing the acute phase reactant item)

At the time of presentation, the mean PVAS was 12.8/63 (range, 6–20); ITAS was 8.5/51 (range, 5–14); ITAS-A was 11.0/57 (range, 6-18). The PVAS showed a strong correlation with both the ITAS (r = 0.77; *P* = 0.002) and ITAS-A (r = 0.84; *P* = 0.002) (Fig. 2). The disease activity scores at first clinic visit are presented in Table 2.

**Fig. 3:**
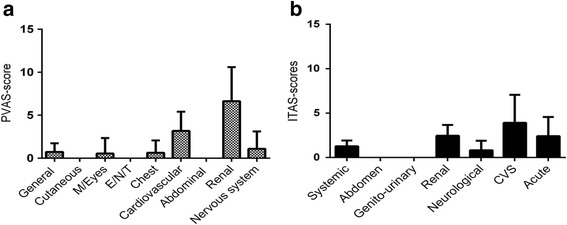
The mean score for each item in the PVAS (**a**) and the ITAS-2010 (**b**). Legend: M/Eyes: mucous membranes and eyes. E/N/T: ear/nose/throat. CVS: cardiovascular system

The average scores for the cardiovascular and renal items were highest. Meanwhile, the abdomen, skin, mucous membranes/eyes, and ENT (ear,nose,throat) all gained a score of 0 (Fig. 3).

### Treatments and outcome

All 11 children were treated with a combination of glucocorticoids (1–2 mg/kg), antihypertensive drugs, and antiplatelet drugs. One patient received intravenous cyclophosphamide pulses, six underwent several rounds of hemodialysis due to renal dysfunction during the hospitalization and follow-up, and two patients underwent angioplasty due to resistant hypertension.

**Fig. 4:**
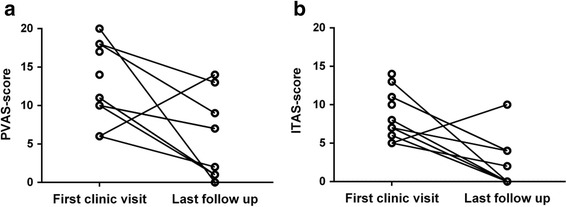
PVAS and ITAS at the first clinic visit and at the last follow-up. Legend: **a** PVAS score before and after therapy. **b** ITAS score before and after therapy

Patiensts were followed up for a median 19 months (range, 2–54 months). The treatment response rate was 77.8% (7/9 patients; two patients were lost to follow-up). Figure 4 shows the changes in scores between first clinic visit and last follow-up.

At last follow-up, patient no. 7 had an increased disease activity score (in both PVAS and ITAS-2010), suggesting no response of treatment. The scores for the other eight patients decreased, although patient no. 1 was still considered to have active disease due to edema and chest tightness.

## Discussion

Pediatric TA is a common form of childhood chronic vasculitis that has various clinical manifestations, including fever, hypertension, kidney damage, and other inflammatory symptoms. Even today, the rate of under diagnosis and misdiagnosis is high due to atypical symptoms.

### Demographic data and clinical presentation

The minimum age of onset was only 1.4 years old, which brings difficulties to our diagnosis. Most doctors would think it was not a rheumatic disease but a congenital disease for the patient’s (no.5) kidney damage. The youngest age reported was 1.5–4 months [3, 9, 10], suggesting that the onset age of TA may be younger than expected. In our cohort, the male-to-female ratio was 1:1.75. The incidence rate and average age of onset were higher in females than males, which is in keeping with the findings of previous studies [3, 9, 10]. However, the incidence in males and the average age of onset have risen in recent years [11]. Gender differences may be related to the role of estrogen or genetics [12]. The average time to diagnosis in our cohort was shorter than that reported previously [3, 9, 10, 13, 14], which may be due to pediatricians’ increased awareness of childhood TA in recent years, as well as regional differences. Tuberculosis infection is thought to be one factor underlying TA [15] and many researchers have reported the co-existence tuberculosis infection in those with TA [3, 14, 15]. The rate of TB infection of our patients was only 22.2%, lower than that in previous reports [3, 13]; this may be related to increased use of tuberculosis vaccines and improvement of medical care.

Here, we found that all cases had hypertension. The most common causes of pediatric hypertension are renal disease, coarctation of the aorta, and renal artery stenosis [16]. Thus, in cases of unexplained hypertension, especially those complicated by elevated levels of acute phase reactants and abnormal urine test results, doctors should monitor patients for BP discrepancies or vessel bruits. Also, when available evidence cannot be explained by organ-specific disease, TA should be considered. We recommend large vessel ultrasound as a screening tool.

### Laboratory and imaging results

TA has no specific immunologic index (e.g., autoantibodies or ANCA) or inflammatory markers [13, 14]. The cohort examined herein was more likely to show an elevated ESR than elevated CRP levels (90% *vs*. 30%, respectively), which is consistent with previous reports [7, 9, 10]. The correlation between acute phase reactant levels and other measurement tools is controversial [3, 7, 10, 17, 18]. Some reports suggest that PVAS shows a better correlation with the ESR than with CRP levels [7]. One Japanese group suggested that an increase in the ESR at first diagnosis can lead to a better prognosis [2]. Here, PVAS showed a stronger correlation with ITAS-A (which includes the acute phase reactants item) than ITAS (which does not), suggesting that the ESR can be used to assess disease activity. However, because the ESR may not be an early indicator of systemic inflammation [7], it should be used in combination with clinical symptoms and imaging results. In recent years, studies have tried to identify acute phase reactants that could replace the ESR and CRP as evaluation indices; these include PTX-3, MMP-9 (matrix metalloproteinase-9), and IL-6 [19–21]. Some studies indicate that PTX-3 is a better indicator of vascular inflammation than CRP. However, multicenter studies are needed to confirm this.

Abnormal lipid metabolism plays a role in the pathogenesis of TA. Some adult TA patients showed dyslipidemia, which may be a risk factor for complicating atherosclerosis [22]. Also, dyslipidemia is suggestive of disease activity [23]. Here, dyslipidemia was identified in 60% (3/5) of cases, similar to that reported previously [22]; however, the disease activity scores for children with and without dyslipidemia were not statistically different. Also, blood lipids do not correlate with CRP and ESR. Thus, it is still unclear whether lipids can be used as a disease activity index for childhood TA.

All tested patients (5 children) had high levels of renin and renal artery stenosis on at least one side, suggesting that elevated renin levels were caused by reduced renal perfusion. This finding is consistent with the literature [13, 24], but it remains unclear whether RAAS can be used to assess the degree of renal artery involvement in TA.

One of 11 patients (9.1%) in our study showed evidence of arterial dissection. It is similar to a Canada study, suggesting the importance of careful vessel wall assessment [25].

Our finding that the most commonly involved vessels were the abdominal aorta and renal arteries is in agreement with previous reports [3, 9, 14]. However, there was no significant difference in the disease activity scores between patients with or without renal artery stenosis. This may be due to different degrees of renal artery stenosis in each patient, and to the small number of cases enrolled.

### Assessment of disease activity

There are multiple tools that can be used to evaluate TA disease activity, including the BVAS and the DEI.Tak (Disease Extent Index-Takayasu); however, there is no recognized standard [26, 27]. Color Doppler ultrasound also has been used to evaluate disease activity yet [18].

PVAS is the special scale for vasculitis activity in children, whereas ITAS-2010 is a TA-specific assessment scale. The results revealed a good correlation between PVAS and ITAS-2010. Here, we found a higher proportion of TA children with renal impairment, which is in agreement with previous reports [9, 13]. The kidneys component of PVAS (12/63) is much more comprehensive than that of ITAS-2010 (3/51), indicating that the former includes a more detailed assessment of renal involvement. Therefore, we recommend that the PVAS scale be used to assess disease activity in TA children with kidney damage.

The rate of mesenteric artery involvement was high (45.5%); however, the abdominal item of PVAS and ITAS scored 0, because there was no evidence of intestinal ischemic pain. Therefore, we emphasize the importance of an overall assessment of the systemic vessels at each follow-up session.

### Advances in treatment of TA

The main medical treatment was glucocorticoids combined with antihypertensive and antiplatelet drugs; this combination showed good efficacy in most cases. For glucocorticoid-resistant children, further immunosuppressive therapy (e.g., cyclophosphamide, azathioprine, or methotrexate) should be considered [28, 29]. Biological agents such as tocilizumab and tumor necrosis factor (TNF) antagonists are used mainly for refractory adult TA cases [30, 31]. TA children who received TNF antagonist therapy showed a satisfactory response in the short term [10, 32, 33], but long-term multicenter studies are lacking.

Blood vessel reconstruction helps to control blood pressure in half of patients with renal artery stenosis [34]. Vascular surgery and interventional surgery can reduce mortality and improve the long-term prognosis for both adults and children, but some patients require a second surgical intervention due to repeat stenosis [35, 36]. Here, two patients with renal artery stenosis had well-controlled blood pressure and TA symptoms after angioplasty, although the long-term prognosis can only be assessed after long-term follow-up.

## Conclusions

The clinical manifestations of childhood TA vary widely. For children with hypertension combined with high levels of inflammatory markers and abnormal urine results, TA should be considered in the differential. Doppler ultrasound scanning of major vessels can be used as a screening tool for early TA diagnosis. PVAS and ITAS-2010 are both helpful for evaluating disease activity in pediatric TA. However, we recommend PVAS as a disease activity assessment tool for patients with kidney damage. Glucocorticoids and antihypertensive agents are effective treatments, and biological agents are being explored. Also, interventional therapy can be an option for those with TA and resistant hypertension.

This is the first study to examine the onset of childhood TA and the use of PVAS and ITAS to monitor disease activity in those with childhood TA in China. However, it should be noted that this study was retrospective in nature and the number of cases was small, raising the possibility of selection bias. Future studies will require a larger sample size and longer-term follow-up to provide more reliable clinical information about pediatric TA.

## Additional files

Additional file 1: Brief comparison of PVAS and ITAS-2010. (DOCX 16 kb)

Additional file 2: Angiographic classification and percentages of 11 TA children. (DOCX 12 kb)

## Abbreviations

ANCA: Anti-neutrophil cytoplasmic antibody
BVAS: Birmingham vasculitis activity score
CTA: Computed tomographic angiography
CRP: C reactive protein
ESR: Erythrocyte sedimentation rate
ENT: Ear/nose/throat
ITAS2010: Indian Takayasu clinical activity score
PVAS: Pediatric vasculitis activity score
RAAS: Renin-angiotensin-aldosterone system
TA: Takayasu arteritis
