# Supplementary material for: Clinical study of children with Takayasu arteritis: a retrospective study from a single center in China
Source: Pediatr Rheumatol Online J. 2017 Apr 17;15:29. doi: 10.1186/s12969-017-0164-2 (PMC5393038; doi:10.1186/s12969-017-0164-2)
Supplement: Supplementary file 1 — Brief comparison of PVAS and ITAS-2010. (DOCX 16 kb) [file 12969_2017_164_MOESM1_ESM.docx]

Additional file 1 Brief comparison of PVAS and ITAS-2010

|  | The contents and scores of the assessment | |
| --- | --- | --- |
|  | PVAS (Max: 63 points) | ITAS-2010 (Max: 51 points) |
| General  /Systemic | Myalgia; Arthralgia or arthritis;  Fever; Weight loss. | Myalgia; Arthralgia; Arthritis;  Fever; Weight loss;  Headache; Malaise. |
|  | (3 points) | (3 points) |
| Cutaneous | Polymorphous exanthema; Livedo; Panniculitis; Skin nodules;  Infarct and so on. | None. |
|  | (6 points) |  |
| Mucous membranes  /Eyes | Mouth or Genital ulcers;  Adnexal or ocular inflammation;  Significant proptosis; Sudden visual loss; Retinal vasculitis. | None. |
|  | (6 points) |  |
| ENT  (ear,nose,throat) | Nasal ulcers; Paranasal sinus or Subglottis involvement;  Conductive or Sensorineural hearing loss. | None. |
|  | (6 points) |  |
| Chest | expiratory dyspnea; pleurisy;  Endobronchial involvement;  Massive haemoptysis/alveolar haemorrhage; Respiratory failure; radiographic changes. | None. |
|  | (6 points) |  |
| Cardiovascular | Loss of pulses; Bruits;  BP discrepancy;  Claudication; Ischaemic cardiac pain;  Congestive cardiac failure;  Valvular heart disease; Pericarditis;  Cardiomyopathy. | New Pulse Loss; Bruits;  Pulse and BP Inequality;  Claudication; Ischemic Cardiac pain; Carotidodynia;  Aortic Incompetence;  Mycardial infarction |
|  | (6 points) | (38 points) |
| Abdominal | Abdominal pain;  Peritonitis; Blood in stools or bloody diarrhea; Bowel ischaemia | Severe abdominal pain. |
|  | (9 points) | (1 points) |
| Renal | Hypertension;  Proteinuria; Haematuria; GFR | Hypertension. |
|  | (12 points) | (3 points) |
| Nervous system | Seizures; Stroke;  Organic confusion/cognitive dysfunction;  Headache; Meningitis/encephalitis;  Cord lesion; Neuropathy. | Seizures; Stroke;  Syncope; Vertigo/dizziness |
|  | (9 points) | (5 points) |
| Genito-urinary | None. | Abortions. |
|  |  | (1 points) |

|  |
| --- |

Calculation of **ITAS-A**: scores for ITAS2010 plus score for acute phase assay as follows:

ITAS -ESR: add 0 for ESR <20, 1 for ESR 21-39, 2 for ESR 40-59, and 3 for ESR >60 mm/Hr.

ITAS-CRP: add 0 for CRP<5, 1 for CRP 6-1, for CRP 11-20, and 3 for CRP >20 mg/dl.
