# Supplementary material for: Clinical study of children with Takayasu arteritis: a retrospective study from a single center in China
Source: Pediatr Rheumatol Online J. 2017 Apr 17;15:29. doi: 10.1186/s12969-017-0164-2 (PMC5393038; doi:10.1186/s12969-017-0164-2)
Supplement: Supplementary file 2 — Angiographic classification and percentages of 11 TA children. (DOCX 12 kb) [file 12969_2017_164_MOESM2_ESM.docx]

| Additional file 2 Angiographic classification and percentages of 11 TA children | | | | |
| --- | --- | --- | --- | --- |
| Types | The involved artery | Numbers(%) | | remarks |
| I | Only branches from the  aortic arch. | 0 | (0%) |  |
| IIa | Ascending aorta, aortic arch, and its branches. | 1 | (9.1%) | 1 P+ |
| IIb | Ascending aorta, aortic arch, and its branches, thoracic descending aorta. | 0 | (0%) |  |
| III | Thoracic descending aorta, abdominal aorta, and/or renal arteries. | 1 | (9.1%) |  |
| IV | Only abdominal aorta and/or renal arteries | 4 | (36.4%) |  |
| V | Combined features of both type  IIb and IV. | 6 | (54.5%) | 1 P+; 1 C+ |
| *C (+) or P (+) stand for the involvement of the coronary or pulmonary artery. | | | | |
